# Aspiration Cytology Diagnosis of Lipoblastoma of the Back

**Published:** 2014-05-21

**Authors:** Sarika Verma, Kalpana Bansal, Vivek Manchanda, Ruchika Gupta

**Affiliations:** 1 Department of Pathology, Chacha Nehru Bal Chikitsalaya, India;; 2 Department of Radiodiagnosis, Chacha Nehru Bal Chikitsalaya, India;; 3 Department of Pediatric Surgery, Chacha Nehru Bal Chikitsalaya, India;

**Dear Sir,**

Lipoblastoma is an unusual benign soft tissue tumor composed of immature white fat. This tumor usually occurs in young children and involves extremities, head and neck, trunk and rarely mediastinum, mesentery and retroperitoneum.[1] Most patients present with asymptomatic gradually enlarging soft tissue mass. Tumours at certain locations may lead to pressure-compression symptoms to the adjacent organs.[1] Magnetic resonance imaging (MRI) is considered the method of choice of radiologic diagnosis of lipoblastoma with characteristic high signal intensity on T1-weighted images.[2] The cytological features of this rare tumor are described only in few reports in the available literature.[3-5]

A six-year-old girl presented with a gradually enlarging painless lump in the right upper back since birth. Local examination revealed a 20 cm x 20 cm soft non-tender lump in the subcutaneous tissues of the back. MRI scans showed a large lobulated mass lesion with predominantly fat intensity on T1-weighted and T2-weighted images. Multiple septae were seen within the lesion. The right inferolateral aspect of the mass showed a small soft tissue component with minimal enhancement on gadolinium contrast (Fig. 1). Fine needle aspiration (FNA) smears showed adipose tissue fragments composed of mature adipocytes as well as lipoblasts with multivacuolated cytoplasm, central indented normochromatic nucleus without nucleoli (Fig. 2a, 2b). A cytologic impression of lipoblastoma was rendered. The child underwent in-toto resection of the tumor. Cut-section of the mass showed a lobulated appearance with yellow and focal grey-white areas. Multiple sections showed a lobulated adipocytic tumor composed predominantly of mature adipocytes separated into lobules by fibrous septae. The septae showed stellate cells and occasional multivacuolated lipoblasts with small central nucleus. No nuclear atypia, mitosis or necrosis was seen (Fig. 2c). Histopathology was consistent with lipoblastoma. The child is doing well one year after surgery.

**Figure:**
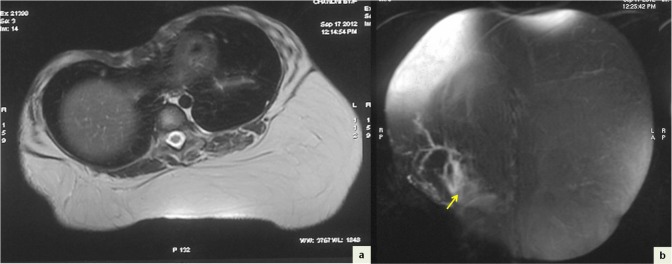
Figure 1: Axial T2-weighted (a) and coronal fat-saturated (STIR) images (b) showing a large lobulated predominantly fat intensity lesion with multiple septae within the subcutaneous tissue of back. A soft tissue component is seen along its right inferolateral aspect (arrow in b).

**Figure:**
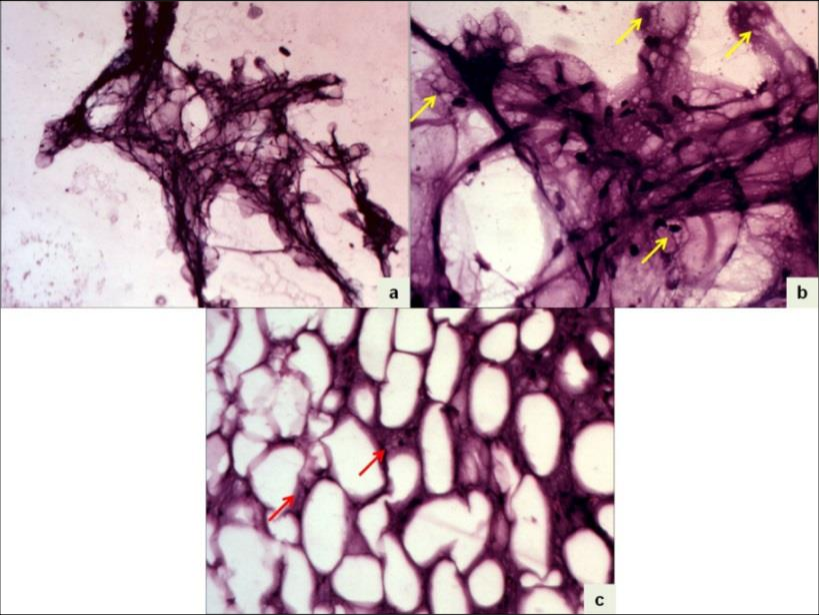
Figure 2: Photomicrographs of the aspiration smear showing adipose tissue fragments (a, Giemsa x40) composed of lipocytes and lipoblasts denoted by arrows (b, Giemsa x400). Histologic section from the excised specimen showing lipoblasts (red arrows) within the septae (c, H/E x400).

There are only few reports of cytologic descriptions of lipoblastoma in the available literature.[3-7] Earlier authors have reported smears with uni- or multi-vacuolated cells with plexiform capillary pattern in a mucoid background. The proportion of lipoblasts and lipocytes vary considerably from case to case.[3,5] In the index case, smears showed tissue fragments composed of mainly lipocytes with few lipoblasts and thin capillaries and a cytologic diagnosis of lipoblastoma could be made. The two main differential diagnoses in cytology of lipoblastoma are myxoid liposarcoma and lipoma. Other entities to be considered include fibrous hamartoma of infancy and other myxoid neoplasms. Myxoid liposarcoma may be indistinguishable from lipoblastoma. The patients' age and location of the tumor can help in this distinction to some extent, since most lipoblastomas occur before three years of age while liposarcomas, usually deep-seated, affect older children and adolescents.[3] Cytologically, myxoid liposarcoma is characterized by myxoid material, small oval cells and branching capillaries. Adipose fragments or lipoblastic differentiation is scarce or absent in most case.[4] The presence of a range of maturation of lipoblasts and lipocytes is characteristic of lipoblastoma on aspiration smears. Surgery is the standard treatment of lipoblastoma. A small risk of local recurrence exists in cases with incomplete resection.[3]

In conclusion, FNA can serve as a useful diagnostic tool in pre-operative diagnosis of lipoblastoma. An accurate cytologic diagnosis can assist the surgeon in making a pre-operative decision.

## Footnotes

**Source of Support:** Nil

**Conflict of Interest:** None declared

